# Convergence and norm estimates of Hermite interpolation at zeros of Chevyshev polynomials

**DOI:** 10.1186/s40064-016-3667-2

**Published:** 2016-11-21

**Authors:** Kamel Al-Khaled, Marwan Alquran

**Affiliations:** Mathematics and Statistics, Jordan University of Science and Technology, P.O. Box 3030, Irbid, 22110 Jordan

**Keywords:** Chevyshev polynomials, Hermite interpolation operator, Norm estimates

## Abstract

In this paper, we investigate the simultaneous approximation of a function *f*(*x*) and its derivative $f^{\prime }(x)$ by Hermite interpolation operator $H_{2n+1}(f;x)$ based on Chevyshev polynomials. We also establish general theorem on extreme points for Hermite interpolation operator. Some results are considered to be an improvement over those obtained in Al-Khaled and Khalil (Numer Funct Anal Optim 21(5–6): 579–588, 2000), while others agrees with Pottinger’s results (Pottinger in Z Agnew Math Mech 56: T310–T311, 1976).

## Background

The main purpose of this paper is to investigate the convergence of Hermite interpolating processes based on the zeros of Chevyshev polynomials. Let $I=[-1,1]$ and $C^p(I)$ be the space consisting of the p-times continuously differentiable real-valued function on *I*. For a given function $f\in C^1(I)$, define its norm by

1 $$ \Vert f \Vert _{1}=\max \left\{ \max _{|x|\le 1} |f(x)|,\max _{|x|\le 1} \left| f^{\prime }(x)\right| \right\}, $$

where $\Vert f\Vert _{1}$ denotes the Chevyshev norm on *I*. For a given nodal matrix $M=\{x_{0}^{(n)},\ldots ,x_{n}^{(n)}\}$, the Hermite interpolation operator $H_{2n+1}(f;x)$ is defined to be the unique polynomial of degree at most $2n+1$ satisfying the conditions: $H_{2n+1}\left( f;x_{i}^{(n)}\right) =f\left( x_{i}^{n}\right)$, and $H^{\prime }_{2n+1}\left( f;x_{i}^{(n)}\right) =f^{\prime }\left( x_{i}^{(n)}\right) , \; i=0,1,2,\ldots ,n$. In what follows, all the functions, weight functions, and spaces of functions under consideration are defined in the interval *I*. Given a positive integer *n*, and a function *f*, we denote by $E_{n}(f)$ the error of best uniform approximation of *f* by algebraic polynomials of degree at most *n*. Pottinger (1976) proved that when the nodal matrix *M* consists of the Chevyshev nodes, the convergence

2 $$ \lim _{n \rightarrow \infty } \left| f- H_{2n+1}(f;.)\right| _{1}=0 $$

holds for $f\in C^2(I)$. There is an extensive literature on Hermite interpolation (see, for example Al-Khaled 2000; Agarwal and Wong 1991; Sababhah et al. 2003, and the references therein) which has been studied from various points of view. Al-Khaled and Khalil (2000) studied the Hermite type interpolation, and gave some estimates for norms of certain interpolation operators on the space of continuously differentiable functions on *I*. The work in Varma and Prasad (1989) deals with mean convergence of $H_{2n+1}(f;x)$ in the special case of interpolation based on zeros of the Chevyshev polynomials. It is proved that $H_{2n+1}(f;x)$ converges in weighted $L^{p}$ norms to $f\in C^1$ at the rate of $E_{2n+1}(f;x)/n$. The Hermite–Fejer interpolating polynomial $F_{2n+1}(f;x)$ is a polynomial of degree at most $2n+1$ that agrees with *f* at the interpolating points, and whose first derivative vanishes there. It is closely related to Hermite interpolating polynomial $H_{2n+1}(f;x)$. Weighted mean convergence of $F_{2n+1}(f;x)$ was studied in details in Nevai and Vertesi (1989), and Nevai and Vertesi (1976). In Nevai and Yuan (1994), the authors consider algebraic polynomials of degree at most $2n-1$ for Hermite interpolation, more precisely, polynomials which interpolate a given function and its derivative at the *n* zeros of the so-called generalized Chevyshev polynomials. The convergence of the polynomials and their first derivatives is studied for weighted $L^p$-norms. Some applications to the Hermite interpolation problem on $[-1,1]$ are illustrated in Berriochoa et al. (2015). In Agarwal (1991), the authors give results for the weighted $L^p$-convergence of derivatives of extended Hermite interpolation on the zeros of Chevyshev polynomials plus additional points. Finally, results on convergence and norm estimation of Hermite and other type of interpolation processes in one or several variables are given in Agarwal and Wong (1993), Costabile and DellAccio (2007), and Mastroianni and Milovanovi (2008), and more recently in the framework of scattered data interpolation (Caira et al. 2012; Costabile et al. 2012, 2013; DellAccio and Tommaso 2016). The aim of this paper is to prove results analogous to (2) using different node system and involving endpoints of the interval too. We shall prove that when the nodal matrix *M* consists of the mixed Chevyshev nodes, the formula (2) also holds for $f\in C^2[-1,1]$. In the last section, we establish a general theorem on extreme points for Hermite operator that agrees with a known result by Pottinger (1976).

## Interpolation with Chevyshev nodes

Let $u_{n}(x)=\frac{\sin ((2n+1)/2)\theta }{\sin \theta /2}$, where $x=\cos \theta$, be the Chevyshev polynomials. We call the zeros of $w(x)=(1-x^2)u_{n}(x): x_{k}^{(n)}=\cos \theta _{k}^{(n)}, \;\theta _{k}^{(n)}=k\pi /n, \; k=0,1,2,\ldots$, the nodes of the Chevyshev polynomials (see, Rivlin 1974). It is clear that

3 $$ w^{\prime }(x)=-2x u_{n}(x)+\left( 1-x^2\right) u_{n}^{\prime }(x), $$

and

4 $$ w^{\prime \prime }(x)=-2 u_{n}(x)-4 x u_{n}^{\prime }(x) +\left( 1-x^2\right) u_{n}^{\prime \prime }(x). $$

Passing a direct calculations, we obtain that

5 $$ w^{\prime }(1)=  -2(1+2n), \quad w^{\prime }(-1)=2(-1)^n$$

6 $$ w^{\prime }(x_{k})= 2(-1)^{k+1}\left[ (1+n)-(2+n) \sin ^2 \theta _{k}/2\right] , \quad (1\le k \le n-1)$$

7 $$ w^{\prime \prime }(1)= -2(2n+1)\left[ \frac{2}{3}n(n+1)+1\right] , \quad w^{\prime \prime }(-1)=(-1)^{n+1}[4n(n+1)+2]$$

8 $$ w^{\prime \prime }\left( x_{k}\right)= (-1)^{k+1}\left[ 2+n\left( (n+3)-\frac{1}{2\sin ^2 \theta _{k}/2}\right) \right] , \quad (1\le k \le n-1). $$

For the sake of convenience, hereafter we replace $x_{k}^{(n)}, \theta _{k}^{(n)}$ by $x_{k}, \theta _{k}$. For $f(x) \in C^1(I)$, the Hermite interpolation operators based on the Chevyshev nodes are

9 $$ H_{2n+1}(f;x)=\sum _{k=0}^nf(x_{k}) v_{k}(x) \ell _{k}^2(x)+\sum _{k=0}^n f'(x_{k})(x-x_{k})\ell _{k}^2(x), $$

where

10 $$\begin{aligned} \ell _{k}(x)= &  a_{k}\frac{w(x)}{(x-x_{k})}, \;\; \left( a_{k}=\frac{1}{w^{\prime }(x_{k})}\right)  \\ v_{k}(x)= &  1-\frac{w^{\prime \prime }(x_{k})}{w^{\prime }(x_{k})}\left( x-x_{k}\right) , \quad 0 \le k\le n. \end{aligned}$$

Using (5)–(8) we can get

11 $$\begin{aligned} a_{k}= &  \left\{ \begin{array}{ll} \frac{-1}{2(2n+1)}, &\quad k=0\\ \frac{(-1)^{k+1}}{2\left( (1+n)-(2+n)\sin ^2 \theta _{k}/2\right) }, &\quad 1 \le k \le n-1\\ \frac{(-1)^{n}}{2}, &\quad k=n, \end{array}\right. \end{aligned}$$

12 $$\begin{aligned} v_{k}(x)= &  \left\{ \begin{array}{ll} 1-\left( \frac{2}{3}n(n+1)+1\right) (x-1), &\quad k=0\\ 1-\frac{2+n\left( (n+3)-\frac{1}{2\sin ^2\theta _{k}/2}\right) }{2\left( (n+1)-(n+2)\sin ^2 \theta _{k}/2\right) }(x-x_{k}),&\quad 1 \le k \le n-1\\ 1+(2n(n+1)+1)(x+1), &\quad k=n, \end{array}\right. \end{aligned}$$

Now, by (10), (11), we have

13 $$\begin{aligned} \ell _{0}(x)= &  \frac{(1+x)\sin \left( \frac{2n+1}{2}\right) \theta }{2(2n+1)\sin \theta /2}, \quad \ell _{n}(x)=\frac{(-1)^{n+1}(1-x)\sin \left( \frac{2n+1}{2}\right) \theta }{2\sin \theta /2} \\ \ell _{k}(x)= &  \frac{(-1)^{k+1}\sin \theta \; \cos \theta /2\quad \sin \left( \frac{2n+1}{2}\right) \theta }{\left( (1+n)-(2+n)\sin ^2\theta _{k}/2\right) (x-x_{k})}, \quad (1 \le k \le n-1), \quad x=\cos \theta . \end{aligned}$$

In view of $|\sin n \theta |\le n |\sin \theta |$, it is clear that

14 $$ |\ell _{0}(x)|\le 1, \quad |\ell _{n}(x)|\le 1, \quad \left( -1\le x \le 1\right). $$

Again by a known result: $\sum\nolimits_{k=1}^{n-1} \ell _{k}^2(x) \le 2$, Rivlin (1974) we get that

15 $$ \left| \ell _{k}(x)\right| \le \sqrt{2} \quad (1 \le k \le n-1). $$

On the other hand, from (3), (6) and $|\sin n \theta |\le n |\sin \theta |$, we have

16 $$\begin{array}{l}\left| {{a_0}{w^\prime }(x)} \right| \le 1,\quad x = \cos \theta \\\left| {{a_n}{w^\prime }(x)} \right| \le 2n + 1,\quad \left| {{a_k}{w^\prime }(x)} \right| \le 4,\quad (1 \le k \le n - 1)\end{array}$$

## Preliminaries

Before proving our main results, the following elementary inequalities will be useful:

### **Lemma 1**

*The following estimates hold for* $a_{k}=\frac{1}{2\left( (n+1)-(n+2)\sin ^2 \theta _{k}/2\right) }=\frac{1}{n+(n+2)\cos \theta _{k}}$

1. $$|a_{k}|\le \frac{1}{n}, \; 1 \le k \le [n/2], or\;\; 0 \le \theta _{k} \le \pi /2$$
2. $$|a_{k}| \le \left| \frac{1}{n-(n+2)n/k}\right| ,\;\; [n/2]+1\le k \le n-1, \;or\; \pi /2<\theta _k<\pi .$$

*Also we shall require.*

### **Lemma 2**

*The following inequality hold true for all* $n \ge 1.$

$$ \frac{n}{10}\cos ^2 \theta _{k}/2\le \left| (n+1)-(n+2) \sin ^2 \theta _{k}/2\right|. $$

*The first result in this paper is*

### **Theorem 1**

*For* $f(x) \in C^1[-1,1],$ *the estimation* $| H_{2n+1}(f;x)| =\mathcal{O}(n^2)$ *holds true as* $n \rightarrow \infty .$

### *Proof*

By (1), (9), it follows that

$$ \left| H_{2n+1}(f;x)\right| \le \Vert f\Vert _{1} \left\{ \sum _{k=0}^n\left| v_{k}(x)\ell _{k}^2(x)\right| +\sum _{k=0}^{n}\left| (x-x_{k})\ell _{k}^2(x)\right| \right\} = \Vert f\Vert _{1}\{J_{1}+J_{2}\} $$

To estimate $J_{1}$, by (10) and (11), it follows that

$$\begin{aligned} \sum _{k=0}^n\left| v_{k}(x) \ell ^2_{k}(x)\right|\le &\,  \left| v_{0}(x) \ell _{0}^2(x)\right| +\sum _{k=1}^{[n/2]}\left| v_{k}(x)\ell _{k}^2(x)\right| \\&+\,\sum _{k=[n/2]+1}^{n-1}\left| v_{k}(x)\ell _{k}^2(x)\right| +\left| v_{n}(x) \ell _{n}^2(x)\right| \\= &  \,J_{1}^{(1)}+J_{1}^{(2)}+J_{1}^{(3)}+J_{1}^{(4)}. \end{aligned}$$

We shall proceed to estimate each of these terms: For $J_{1}^{(1)}$, by (14), we have

$$\begin{aligned} J_{1}^{(1)}=\,\left| v_{0}(x) \ell _{0}^2(x)\right|\le\, &\,  \ell _{0}^2(x)+\left( \frac{2}{3}n(n+1)+1\right) (1-x^2)\frac{(1+x^2)\sin ^2\left( \frac{2n+1}{2}\right) \theta }{4(2n+1)^2\sin ^2 \theta /2}\\\le &\,  1+\frac{1}{12}(2n+1)^2\frac{\left( 4 \sin ^2 \theta /2 \cos ^2 \theta /2\right) \left( 2 \cos ^2 \theta /2\right) \sin ^2\left( \frac{2n+1}{2}\right) \theta }{(2n+1)^2\sin ^2 \theta /2}\le \frac{5}{3}. \end{aligned}$$

For $J_{1}^{(4)}$, we have

$$\begin{aligned} J_{1}^{(4)}=\left| v_{n}(x) \ell _{n}^2(x)\right|\le\, &\,  \ell _{n}^2(x)+\{2n(n+1)+1\}(1+x)\frac{(1-x^2)\sin ^2(2n+1/2)\theta }{4\sin ^2 \theta /2}\\\le &\,  1+\left( 4n^2+4n+2\right) =4n^2+4n+3. \end{aligned}$$

For $J_{1}^{(2)}$, we have

$$\begin{aligned} J_{1}^{(2)}= \sum _{k=1}^{[n/2]}\left| v_{k}(x)\ell _{k}^2(x)\right|\le &\,  \sum _{k=1}^{[n/2]}\left| \ell _{k}^2(x)\right| +\sqrt{2}\sum _{k=1}^{[n/2]}\left| \frac{a_{k}w(x)\left( 2+n(n+3)-\frac{1}{2\sin ^2 \theta _{k}/2}\right) }{2\left( (n+1)-(n+2)\sin ^2\theta _{k}/2\right) }\right| \\\le &\,  2+2\sqrt{2}\sum _{k=1}^{[n/2]}\left| \frac{2(n+1)(n+2)\sin ^2 \theta _{k}/2-n}{4\left( (n+1)-(n+2)\sin ^2\theta _{k}/2\right) ^2 \sin ^2\theta _{k}/2}\right| , \end{aligned}$$

where in the last inequality we used $|w(x) \le 2$. Now using the first part of Lemma 1, and $\sin ^2\theta _{k}/2=\sin ^2\theta _{k}/(4\cos ^2 \theta _{k}/2)$, we obtain

$$\begin{aligned} J_{1}^{(2)}&= 2+2\sqrt{2}\sum _{k=1}^{[n/2]}\left| \frac{4 \cos ^2 \theta _{k}/2\left( 2(n+1)(n+2)+n\right) }{n^2 \sin ^2 \theta _{k}}\right| \\&\le \,  2+2\sqrt{2}\sum _{k=1}^{[n/2]}\left| \frac{4\left( 2(n+1)(n+2)+n\right) }{n^2\sin ^2\theta _{k}}\right| . \end{aligned}$$

Using the Jordan inequality: $\sin \theta \ge 2\theta /\pi , \; 0 \le \theta \le \pi /2$, we have

$$\begin{aligned} J_{1}^{(2)}\le &  2+2\sqrt{2}\sum _{k=1}^{[n/2]}\left| \frac{4(2(n+1)(n+2)+n)n^2}{4n^2k^2}\right| \\\le &  2+2\sqrt{2} \left( 2n^2+7n+4\right) \sum _{k=1}^{[n/2]}\frac{1}{k^2}\\\le &  2+2\sqrt{2} \left( 2n^2+7n+4\right) \sum _{k=1}^{\infty }\frac{1}{k^2}\le 2+2\sqrt{2} \left( 2n^2+7n+4\right) \frac{\pi ^2}{3}. \end{aligned}$$

For $J_{1}^{(3)}$, we have

$$\begin{aligned} J_{1}^{(3)}= &  \sum _{\left[ \frac{n}{2}\right] +1}^{n-1}\left| v_{k}(x)\ell _{k}^2(x)\right| \\\le &  \sum _{k=\left[ \frac{n}{2}\right] +1}^{n-1} \ell _{k}^2(x)+\sqrt{2}\sum _{k=\left[ \frac{n}{2}\right] +1}^{n-1}\left| \frac{a_{k} w(x)\left( 2+n\left( (n+3)-\frac{1}{2\sin ^2 \theta _{k}/2}\right) \right) }{2\left( (n+1)-(n+2) \sin ^2 \theta _{k}/2\right) }\right| \\\le &  2+2 \sqrt{2}\sum _{k=1}^{\left[ \frac{n}{2}\right] }\left| \frac{2(n+1)(n+2)\sin ^2 \theta _{k}/2-n}{4\left( (n+1)-(n+2)\sin ^2\theta _{k}/2\right) ^2 \sin ^2\theta _{k}/2}\right| . \end{aligned}$$

Now by the second part of Lemma 1, and $\sin ^2\theta _{k}/2=\sin ^2 \theta _{k}/\left( 4 \cos ^2\theta _{k}/2\right)$ we obtain

$$\begin{aligned} J_{1}^{(3)}\le \sum _{k=\left[ \frac{n}{2}\right] +1}^{n-1}\left| \frac{4(2(n+1)(n+2)+n)\cos ^2\theta _{k}/2}{n^2\left( \frac{k-(n+2)}{k}\right) ^2 \sin ^2\theta _{k}}\right| . \end{aligned}$$

Since $\sin \theta _{k}=\sin \theta _{k-n}$, we can apply Jordan inequality again, so that

$$\begin{aligned} J_{1}^{(3)}\le\, &  2+2\sqrt{2}\sum _{k=\left[ \frac{n}{2}\right] +1}^{n-1}\left| \frac{4\left( 2(n+1)(n+2)+n\right) n^2}{4n^2\left( \frac{k-(n+2)}{k}\right) ^2k^2}\right| \\\le \,&  2+2 \sqrt{2}\sum _{k=\left[ \frac{n}{2}\right] +1}^{n-1}\left| \frac{2(n+1)(n+2)+n}{(k-(n+2))^2}\right| . \end{aligned}$$

But,

$$\begin{aligned} \sum _{k=\left[ \frac{n}{2}\right] +1}^{n-1}\frac{1}{\left( k-(n+2)\right) ^2}=\sum _{k=\left[ \frac{n}{2}\right] +1}^{-3}\frac{1}{k^2} =\sum _{k=3}^{\left[ \frac{n}{2}\right] +1}\frac{1}{k^2}\le \frac{\pi ^2}{6}. \end{aligned}$$

Therefore,

$$ J_{1}^{(3)}\le 2+ \frac{\pi ^2\sqrt{2}}{3}\left( 2n^2+7n+4\right). $$

Combining the estimates of $J_{1}^{(1)},J_{1}^{(2)},J_{1}^{(3)}$ and $J_{1}^{(4)}$, there is a constant $K_{1}$ such that

17 $$ J_{1}\le K_{1}\left( 11+22n+5n^2\right). $$

To estimate $J_{2}$, by (13), (15) and Lemma 2, we have

$$\begin{aligned} \sum _{k=1}^{n-1}\left| (x-x_{k})\ell _{k}^2(x)\right|= &  \sum _{k=1}^{n-1}\sqrt{2}\left[ \frac{w(x)}{2(n+1)-(n+2)\sin ^2\theta _{k}/2}\right] \\\le &  \sum _{k=1}^{n-1}\left| \frac{10 \sqrt{2}}{n \cos ^2 \theta _{k}/2}\right| \le 40 \sqrt{2} \sum _{k=1}^{n-1}\frac{1}{n \sin ^2 \theta _{k}}. \end{aligned}$$

Because

$$\begin{aligned} \sum _{k=1}^{n-1}\frac{1}{n\sin ^2 \theta _{k}}=\sum _{k=1}^{\left[ \frac{n}{2}\right] }\frac{1}{\sin ^2 \theta _{k}} +\sum _{k=1}^{n-\left[ \frac{n}{2}\right] -1}\frac{1}{\sin ^2 \theta _{n-k}} \end{aligned}$$

and $\sin \theta _{n-k}=\sin \theta _{k}$, using the Jordan inequality: $\sin \theta \ge 2\theta /\pi$ for $0 \le \theta \le \pi /2$, we obtain

$$\begin{aligned} \sum _{k=1}^{n-1}\frac{1}{n\sin ^2 \theta _{k}}\le \sum _{k=1}^{\left[ \frac{n}{2}\right] }\frac{n}{4k^2}+\sum _{k=1}^{n-\left[ \frac{n}{2}\right] -1}\frac{n}{4k^2} <\frac{n}{2}\sum _{k=1}^{\infty }\frac{1}{k^2}=\frac{\pi ^2n}{12}. \end{aligned}$$

Therefore,

$$ \sum _{k=1}^{n-1}\left| (x-x_{k})\ell _{k}^2(x)\right| \le \frac{\sqrt{2}\pi ^2}{3}n. $$

Again, by (13), we have $|(x-1)\ell _{0}^2(x)|+|(x+1)\ell _{n}^2(x)|\le |x-1|+|x+1|=2, \; (-1<x<1)$. Therefore,

18 $$ J_{2} \le 50 n+2. $$

From this and (17), we obtain the desired result. $\square$

### **Theorem 2**

*For* $f(x) \in C^1[-1,1]$ *, the estimation* $|H^{\prime }_{2n+1}(f;x)| =\mathcal{O}(n^2)$ *holds true as* $n\rightarrow \infty$.

### *Proof*

By (9) and $\frac{\hbox {d}}{\hbox {d}x}\sum _{k=0}^n (v_{k}(x) \ell _{k}^2(x))=0$, we have

$$\begin{aligned} H^{\prime }_{2n+1}(f;x)= &  \sum _{k=0}^{n}\left( f(x_{k})-f(x)\right) \left( v_{k}(x)\ell _{k}^2(x)\right) ^{\prime }+\sum _{k=0}^{n}f^{\prime }(x_{k})\left( (x-x_{k})\ell _{k}^2(x)\right) ^{\prime }\\= &  \sum _{k=0}^n\left( f(x_{k})-f(x)\right) \left( v^{\prime }_{k}(x)\ell ^2_{k}(x)+2v_{k}(x)\ell _{k}(x)\ell ^{\prime }_{k}(x)\right) \\&+\,\sum _{k=0}^{n}f^{\prime }(x_{k})\left( \ell ^2_{k}(x) +2(x-x_{k})\ell _{k}(x)\ell ^{\prime }_{k}(x)\right) . \end{aligned}$$

Since $|f(x)-f(x_{k})|\le \max _{|x|\le 1}|f^{\prime }(x)||x-x_{k}|$, the above formula implies that

$$\begin{aligned} \left| H^{\prime }_{2n+1}(f;x)\right|\le &  \max _{|x|\le 1} |f^{\prime }(x)|\left\{ \sum _{k=0}^n \left| (x-x_{k})v^{\prime }_{k}(x)\ell ^2_{k}(x)\right| +\sum _{k=0}^n \ell ^2_{k}(x)\right. \\&\left. +\,2\sum _{k=0}^n\left| (x-x_{k})v_{k}(x)\ell _{k}(x)\ell ^{\prime }_{k}(x)\right| +2\sum _{k=0}^n\left| (x-x_{k})\ell _{k}(x)\ell ^{\prime }_{k}(x)\right| \right\} \\= &  \max _{|x|\le 1} |f^{\prime }(x)|\left\{ I_{1}+I_{2}+2I_{3}+2I_{4}\right\} . \end{aligned}$$

For $I_{1}$, by (12), we know that

$$\begin{aligned} I_{1}&= \,  \left| (x-1)\left( \frac{2}{3}n(n+1)+1\right) \ell _{0}^2(x)\right| +\left| (x+1)(2n(n+1)+1)\ell _{n}^2(x)\right| \\&+\,\sum _{k=1}^{\left[ \frac{n}{2}\right] }\left| (x-x_{k})\left( \frac{2+n\left( (n+3)-\frac{1}{2\sin ^2\theta _{k}/2}\right) }{2\left( (n+1)-(n+2)\sin ^2\theta _{k}/2\right) }\right) \ell ^2_{k}(x)\right| \\&+\,\sum _{k={\left[ \frac{n}{2}\right] }+1}^{n-1}\left| (x-x_{k})\left( \frac{2+n\left( (n+3)-\frac{1}{2\sin ^2\theta _{k}/2}\right) }{2\left( (n+1)-(n+2)\sin ^2\theta _{k}/2\right) }\right) \ell ^2_{k}(x)\right| \\&= \,  I_{1}^{(1)}+I_{1}^{(2)}+I_{1}^{(3)}+I_{1}^{(4)}. \end{aligned}$$

Again by (13), we have

$$\begin{aligned} I_{1}^{(1)}= &\,  \left| (1-x)\left( \frac{2}{3}n(n+1)+1\right) +\frac{(1+x)^2\sin ^2\left( \frac{2n+1}{2}\right) \theta }{4(2n+1)^2\sin ^2\theta /2}\right| \\\le &  \left| \frac{1}{3}(2n^2+2n+1)\frac{2}{(2n+1)^2}\right| \le \frac{1}{3}. \end{aligned}$$

Similarly, we can show that $I_{1}^{(2)}\le 4n^2+4n+2.$ For $I_{1}^{(3)}$, with the aid of the first part of Lemma 1, we have

$$\begin{aligned} I_{1}^{(3)}= &\,  \sum _{k=1}^{\left[ \frac{n}{2}\right] }\left| (x-x_{k})\left( \frac{2(n+1)(n+2)\sin ^2\theta _{k}/2-n}{4\left( (n+1)- (n+2)\sin ^2\theta _{k}/2\right) \sin ^2\theta _{k}/2}\right) \ell ^2_{k}(x)\right| \\\le &  \sqrt{2}\sum _{k=1}^{\left[ \frac{n}{2}\right] }\left| \frac{2(n+1)(n+2)+n}{4\left( (n+1)- (n+2)\sin ^2\theta _{k}/2\right) ^2\sin ^2\theta _{k}/2}\right| \\\le &  \sqrt{2}\sum _{k=1}^{\left[ \frac{n}{2}\right] }\left| \frac{2(n+1)(n+2)+n}{n^2\sin ^2\theta _{k}/2}\right| \le \sqrt{2}\left( 2n^2+7n+4\right) \sum _{k=1}^{\infty }\frac{1}{k^2}\\\le &  \sqrt{2}\left( 2n^2+7n+4\right) \frac{\pi ^2}{6}. \end{aligned}$$

For $I_{1}^{(4)}$, with the aid of the second part of Lemma 1, we have

$$\begin{aligned} I_{1}^{(4)}\le &  \sum _{k=\left[ \frac{n}{2}\right] +1}^{n-1}\left| \frac{2(n+1)(n+2)+n}{4\left( (n+1)-(n+2)\sin ^2\theta _{k}\right) ^2\sin ^2\theta _{k}/2}\right| \\\le &  \sqrt{2}\sum _{k=\left[ \frac{n}{2}\right] +1}^{n-1}\left| \frac{4(2(n+1)(n+2)+n)\cos ^2\theta _{k}/2}{n^2\sin ^2\theta _{k}\left( \frac{k-(n+2)}{k}\right) ^2}\right| . \end{aligned}$$

Since $\sin \theta _{k}=\sin \theta _{k-n}$, we can apply Jordan inequality to obtain

$$\begin{aligned} I_{1}^{(4)}\le &  \sqrt{2}\sum _{k=\left[ \frac{n}{2}\right] +1}^{n-1}\left| \frac{2(n+1)(n+2)+n}{(k-(n+2))^2}\right| \\= &\,  \sqrt{2}(2(n+1)(n+2)+n)\sum _{k=\left[ \frac{n}{2}\right] +1}^{n-1}\frac{1}{(k-(n+2))^2} =\sqrt{2}(2(n+1)(n+2)+n)\sum _{k=3}^{\left[ \frac{n}{2}\right] +1}\frac{1}{k^2}\\\le &  \sqrt{2}(2(n+1)(n+2)+n) \frac{\pi ^2}{6}. \end{aligned}$$

Summarizing these estimates for $I_{1}$, we get $I_{1}\le \mathcal{O}(n^2), (n \rightarrow \infty )$. For $I_{2}$, using the fact that $I_{2} =\sum _{k=1}^{n-1} \ell _{k}^2(x) \le 2$ and (2.12) we get $I_{2} \le 4$. For $I_{3}$,

$$\begin{aligned} I_{3}&= \,  \sum _{k=0}^{n} \left| (x-x_{k}) v_{k}(x) \ell _{k}(x) \ell ^{\prime }_{k}(x)\right| \\\le &  \sum _{k=0}^n\left| v_{k}(x) \ell _{k}(x)\left\{ a_{k} w^{\prime }(x)-\ell _{k}(x)\right\} \right| \\\le &  \left| v_{0}(x) \ell _{0}(x) a_{0} w^{\prime }(x)\right| +\left| v_{n}(x) \ell _{n}(x) a_{n} w^{\prime }(x)\right| +\left| v_{0}(x)\ell ^2_{0}(x)\right| +\left| v_{n}(x)\ell ^2_{n}(x)\right| \\&+ \,\sum _{k=1}^{\left[ \frac{n}{2}\right] }\left| v_{k}(x) \ell _{k}(x) a_{k} w^{\prime }(x)\right| +\sum _{k=\left[ \frac{n}{2}\right] +1}^{n-1}\left| v_{k}(x) \ell _{k}(x) a_{k} w^{\prime }(x) \right| +\sum _{k=1}^{\left[ \frac{n}{2}\right] }\left| \ell _{k}^2(x) v_{k}(x) \right| \\&+\,\sum _{k=\left[ \frac{n}{2}\right] +1}^{n-1}\left| \ell _{k}^2(x) v_{k}(x) \right| \\&= \,  I_{3}^{(1)}+I_{3}^{(2)}+I_{3}^{(3)}+I_{3}^{(4)}+I_{3}^{(5)}+I_{3}^{(6)}+I_{3}^{(7)}+I_{3}^{(8)}. \end{aligned}$$

Note that $|a_{0} w^{\prime }(x)|\le 1$, so that

$$\begin{aligned} I_{3}^{(1)}\le &  \left| \ell _{0}(x)\left( 1-\left( \frac{2}{3}n(n+1)+1\right) \right) (x-1)\right| \\\le &  \left| \ell _{0}(x) \right| +\left| \left( \frac{2}{3}n(n+1)+1\right) \frac{\left( 1-x^2\right) \sin \left( \frac{2n+1}{2}\right) \theta }{2(2n+1)\sin \theta /2}\right| \le 1+\frac{1}{3}(n+1), \end{aligned}$$

and

$$\begin{aligned} I_{3}^{(2)}\le &  \sqrt{2}(2n+1)+\left| (2n(n+1)+1)(x+1)\frac{(1-x^2)(1+x)\sin ^2\left( \frac{2n+1}{2}\right) \theta }{2(1+x)\sin \theta /2} \right| \\\le \,&  2n^2+7n+3. \end{aligned}$$

For $I_{3}^{(3)}$, we have,

$$\begin{aligned} I_{3}^{(3)}\le \ell _{0}^2(x)+\left( \frac{2}{3}n(n+1)+1\right) (1-x)\frac{(1+x)^2\sin ^2\left( \frac{2n+1}{2}\right) \theta }{2(1+x)\sin \theta /2}\le \frac{5}{3}. \end{aligned}$$

Similar to the estimate in $I_{3}^{(2)}$, we can show that $I_{3}^{(4)}=|v_{n}(x) \ell _{n}^2(x)|\le 4n^2+4n+3.$ For $I_{3}^{(5)}$: By using $|w^{\prime }(x)|\le 2(2n+1)$ and part one of Lemma 1, we obtain

$$\begin{aligned} I_{3}^{(5)}\le \,&  4\sum _{k=1}^{\left[ \frac{n}{2}\right] }2(2n+1)\left( \frac{2+n(n+3)-\frac{n}{\sin ^2 \theta _{k}/2}}{8((n+1)-(n+2)\sin ^2 \theta _{k}/2)^3}+\ell _{k}(x)\right) \\\le\, &  2(2n+1)\left( n\left( \sum _{k=1}^{\left[ \frac{n}{2}\right] }\ell _{k}^2(x)\right) ^{1/2}+\sum _{k=1}^{\left[ \frac{n}{2}\right] }\frac{2(n+1)(n+2)+n}{n^3\sin ^2\theta _{k}/2}\right) \\\le \,&  2(2n+1)\left( \sqrt{2}n +\sum _{k=1}^{\left[ \frac{n}{2}\right] }\frac{n^2(2(n+1)(n+2)+n)}{n^3k^2}\right) \\\le \,&  \left( 4\sqrt{2}+4\pi ^2/3\right) n^2 +\left( 2\sqrt{2}+4\pi ^2\right) n +5 \pi ^2 /3. \end{aligned}$$

For $I_{3}^{(6)}$, we have

$$\begin{aligned} I_{3}^{(6)}\le &  \,2(2n+1)\left( \sum _{k=\left[ \frac{n}{2}\right] +1}^{n-1}\left( \frac{2(n+1)(n+2)\sin ^2 \theta _{k}/2-n}{8((n+1)-(n+2)\sin ^2\theta _{k}/2)^3 \sin ^2\theta _{k}/2}+\ell _{k}(x)\right) \right) \\\le &\,  2(2n+1)\left( n\left( \sum _{k=\left[ \frac{n}{2}\right] +1}^{n-1}\ell _{k}(x)\right) ^{1/2}+\sum _{k=\left[ \frac{n}{2}\right] +1}^{n-1}\left| \frac{2(n+1)(n+2)\sin ^2\theta _{k}/2-n}{8\left( (n+1)-(n+2)\sin ^2\theta _{k}/2\right) ^3\sin ^2\theta _{k}/2}\right| \right) \\\le &\,  2(2n+1)\left( \sqrt{2}n+\sum _{k=\left[ \frac{n}{2}\right] +1}^{n-1}\left| \frac{20(n+1)(n+2)\sin ^2\theta _{k}/2-10n}{n\cos ^2\theta _{k}/2 \sin ^2\theta _{k}/2(n-n(n+2)/k)^2}\right| \right) \\\le &\,  2(2n+1)\left( \sqrt{2}n+\sum _{k=\left[ \frac{n}{2}\right] +1}^{n-1}\frac{40k^2(2(n+1)(n+2)\sin ^2\theta _{k}/2-n)}{n^3\sin ^2\theta _{k}(k-(n+2))^2}\right) . \end{aligned}$$

Since $\sin {\theta _{k}}=\sin \theta _{k-n}$, we can apply Jordan inequality to obtain

$$ I_{3}^{(6)}\le 2(2n+1)\left( \sqrt{2}n+\sum _{k=\left[ \frac{n}{2}\right] +1}^{n-1}\frac{10\left( 2n^2+7n+3\right) }{\pi ^2 n(k-(n+2))^2}\right) \le 2(2n+1)\left( \sqrt{2}n+\frac{20n+70}{6}\right). $$

It is easy to show that $I_{3}^{(7)}\le 2+ \frac{\pi ^2\sqrt{2}}{3}(2n^2+7n+4)$ and $I_{3}^{(8)}\le 2 +K_{2}(2n^2+7n+4)$ for some constant $K_{2}$. Combining all the estimates for $I_{3}^{(1)}$ till $I_{3}^{(8)}$, we observe that $\parallel I_{3} \parallel ={{\mathcal {O}}}(n^2)$. For $I_{4}$:

$$ I_{4}\le \sum _{n=0}^n \ell _{k}^2(x)+\sum _{k=0}^n |a_{k}w^{\prime }(x)\ell _{k}(x)|. $$

In view of $\sum _{k=0}^n \ell _{k}^2(x) \le 4$; and using Lemma 2, we have

$$\begin{aligned} \sum _{k=0}^n |a_{k}w^{\prime }(x)\ell _{k}(x)|\le &\,  |a_{0}w'(x) \ell _{0}(x)|+\sum _{k=0}^{n-1}|a_{k}w'(x)\ell _{k}(x)|+|a_{n}w'(x)\ell _{n}(x)|\\\le &  \,1+\sqrt{2}\sum _{k=1}^{n-1}\frac{2(2n+1)}{2((n+1)-(n+2)\sin ^2\theta _{k}/2)}+2n+1\\\le &\,  2+2n+2\sqrt{2}(2n+1)\sum _{k=1}^{n-1}\frac{5}{n\cos ^2 \theta _{k}/2}\\\le &  2+2n+40 \sqrt{2}(2n+1)\left( \frac{n \pi ^2}{12}\right) . \end{aligned}$$

Therefore,

$$ I_{4}\le 6+\left( 2+\frac{10 \sqrt{2}\pi ^2}{3}\right) n+\left( \frac{20\sqrt{2}\pi ^2}{3}\right) n^2. $$

Combining all the estimates of $I_{1}, I_{2}, I_{3}, I_{4}$, we complete the proof of Theorem 2. $\square$

## The main convergence result

The norm estimates obtained in the previous section are used to prove convergence of the interpolation functions in the $C^2$-norm, and that error estimates are given in terms of the modulus of continuity of the second derivative. Before we state our theorem, we define $E_{n}^1(f)=\inf _{p\in H_{n}} \Vert f-p\Vert _{1}$ where ${{\mathcal {P}}}_{n}$ is the space of all polynomials of degree $\le n$. (this definition is stated in Pottinger 1978, p. 272).

### **Theorem 3**

*If* $f(x) \in C^2[-1,1]$ *and* $\omega (f^{(2)};\delta )$ *is the modulus of continuity of* $f^{(2)}$ *, then*

$$ \Vert H_{2n+1}-f\Vert _{1} \le {\bar{M}}\omega \left( f^{(2)};1/n\right) \;\; (n\ge 2). $$

*Hereafter* ${\bar{M}}$ *denote different constants which are independent of n*.

### *Proof*

Since Hermite interpolation is a projection operator, we have the standard inequality

$$ \Vert H_{2n+1}(f;.)-f\Vert _{1} \le (1+\Vert H_{2n+1}\Vert _{1})E_{2n+1}(f) $$

where the term $E_{2n+1}(f)=\min \{\Vert f-p \Vert _{1} \; | \; p \in \mathcal{P}_{n+1}(f)\}$ and $H_{k}$ is the space of all polynomials of degree at most *k*. Using Theorems 1 and 2, we have

$$ |H_{2n+1}(f;x)-f(x)|\le {\bar{M}} n^2 E_{2n+1}^1(f), \;\;|H^{\prime }_{2n+1}(f;x)-f^{\prime }(x)|\le {\bar{M}} n^2 E_{2n+1}^1(f) $$

here $E^1_{n}(f)=\inf _{p\in H_{n}}\parallel f-p\parallel _{1}$. By (1.1) and the formula $E_{n}^2(f)=E_{n-2}(f^{(2)})$ (see, Pottinger 1978, p. 272),where $E_{n-2}(f^{(2)})$ is the best approximation to $f^{(2)}$, we have

$$ \Vert H_{2n+1}(f;.)-f\Vert _{1}\le M n^2 E_{2n-1}\left( f^{(2)}\right) $$

In view of $f\in C^2[-1,1]$, using Jackson’s theorem (Natanson 1965, p. 86), we have

$$ E_{2n-1}\left( f^{(2)}\right) \le \frac{M}{n^2}\omega \left( f^{(2)};1/n\right) , \; n \ge 2 $$

which completes the proof of Theorem 3. $\square$

## Results of some interpolation operators

Let *f* be a continuously differentiable function on $I=[-1,1]$. Let $C^p(I)$ be the space consisting of the $p-$times continuously differentiable real-valued functions on *I*. For $f \in C(I)$, define $\Vert f\Vert _{\infty }=\sup _{x\in I}|f(x)|.$ Set $X=C(I) \times C(I)$ with the usual operations

- $$(f_{1},g_{1})+(f_{2},g_{2})=(f_{1}+g_{1},f_{2}+g_{2})$$, for all $$f_{i}, g_{i}\in C(I), i=1,2.$$
- $\lambda (f,g)=(\lambda f,\lambda g)$, where $\lambda \in$ field, and $f, g\in C(I)$

Then *X* is a vector space. For $(f,g) \in X$, one can define different types of norms. We are interested in two norms:

19 $$\begin{aligned} \Vert (f, g) \Vert _{\infty }= &\,  \max \left\{ \Vert f\Vert _{\infty },\Vert g\Vert _{\infty }\right\} \end{aligned}$$

20 $$\begin{aligned} \Vert (f,g) \Vert _{p}= &\,  \left( \Vert f\Vert _{\infty }^p+\Vert g\Vert _{\infty }^p\right) ^{1/p}, \quad 1 \le p < \infty . \end{aligned}$$

It is known that *X* is a Banach space under such norms. Let ${{\mathcal {P}}}_{2n+1}$ denote the space of all polynomials of degree less than or equal to $2n+1$. Now define the operator

21 $$\begin{aligned}&H_{n} : C(I) \times C(I) \rightarrow {{\mathcal {P}}}_{2n+1} \\&H_{n}(f, g)(x)=\sum _{i=0}^n v_{i}(x)\ell ^2_{i}(x)f(x_{i})+\sum _{i=0}^n(x-x_{i})\ell ^2_{i}(x)g(x_{i}), \end{aligned}$$

where $x_{i}$ are some nodes on *I*, and $v_{i}(x), \ell _{i}(x)$ are functions associated with some weight function *w*(*x*) corresponding to the nodes $x_{i}'s$. Let

$$\begin{aligned} \ell _{i}(x)=\frac{\prod _{i=0}^n (x-x_{j})}{\prod _{j=0}^n (x_{i}-x)}, \end{aligned}$$

and

$$\begin{aligned} v_{i}(x)=1-\frac{w^{\prime \prime }(x)}{w^{\prime }(x)}(x-x_{i}), \end{aligned}$$

where, $w(x)=\prod _{i=0}^n (x_{i}-x_{j}).$ The operator $H_{n}$ is the same as the Hermite interpolation operator if $g=f^{\prime }$ in Eq. (21). Al-Khaled and Khalil (2000), also Pottinger (1976), they studied the Hermite type interpolation and gave some estimates for norms of certain interpolation operators on the space of continuously differentiable functions on *I*. In this section, we study the Hermite interpolation operator given by Eq. (21), and establish general theorem on extreme points of some interpolation operators. The following known result will be needed in this paper.

### **Lemma 3**

*Let* $\{x_{1}, x_{2},\ldots ,x_{k}\} \subset I$ *be pairwise distinct real numbers and let* $y_{1}, y_{2},\ldots ,y_{k} \subset R$ *be arbitrary real numbers. Then there exists* $f\in C(I)$ *such that* $f(x_{i})=y_{i}$.

### *Proof*

The points $(x_{i}, y_{i})$ can be connected, in order with their indices, by a polygonal chain, which is the graph of a continuous function provided that $x_{1}<x_{2}<\cdots <x_{n}.$ $\square$

Note that $H_{n}$ defined as in Eq. (21) is a finite rank operator. Further:

$$\begin{aligned} \Vert H_{n} \Vert =\sup \left\{ \Vert H_{n}(f,g)\Vert _{\infty }, (f,g) \in {{\mathcal {B}}}(X)\right\} \end{aligned}$$

where ${{\mathcal {B}}}(X)$ is the unit ball of *X* under the norm (19). Now $(f(x_{0}),f(x_{1}),\ldots ,f(x_{n}))\in {\mathbf{R}}^{n+1}$, where ${\mathbf{R}}^{n+1}=\{(\alpha _{0},\alpha _{1},\ldots ,\alpha _{n}): \alpha _{i} \in {\mathbf{R}} \}$, and for $z\in {\mathbf{R}} ^{n+1}$ we have $\Vert z\Vert _{\infty }=\sup _{0 \le i \le n}|z_{i}|$. Similarly for $(g(x_{0}),g(x_{1}),\ldots ,g(x_{n}))$. By Lemma 3, we have

### **Lemma 4**

$\{f(x_{i}): f \in C(I) \}={\mathbf{R}}^{n+1}$ *, where equality is isometry equality.*

By Lemma 3, we obtain

$$\begin{aligned} \Vert H_{n}\Vert =\sup \Vert \sum _{i=0}^{n}v_{i}(x) \ell _{i}^2(x) a_{i}+\sum _{i=0}^n (x-x_{i})\ell _{i}^2(x)b_{i}\Vert _{\infty } \end{aligned}$$

where the supremum is taken over all $(a_{i}), (b_{i})$ in the unit ball of ${\mathbf{R}}^{n+1}$. Hence we can look at $H_{n}$ as an operator

$$\begin{aligned} H_{n}: {\mathbf{R}}^{n+1}\times {\mathbf{R}}^{n+1} \rightarrow {{\mathcal {P}}}_{2n+1}. \end{aligned}$$

Let $Y={\mathbf{R}}^{n+1}\times {\mathbf{R}}^{n+1}$, then Y is a finite dimensional normed space. Hence $H_{n}$ attains its norm at some point in ${{\mathcal {B}}}(Y)$. Again ${{\mathcal {P}}}_{2n+1}$ is finite dimensional, thus ${{\mathcal {P}}}_{2n+1}$ is reflexive, so $\mathcal{P}_{2n+1}=\left[ ({{\mathcal {P}}}_{2n+1})^{*}\right] ^{*}$. Let $G=\mathcal{P}_{2n+1}^{*}$. From the theory of tensor products of Banach spaces (see, Khalil 1990), we have

$$\begin{aligned} (E{\hat{\otimes }} F)^{*}=L(E, F^{*}). \end{aligned}$$

Taking $E=Y, F={{\mathcal {P}}}_{2n+1}^{*}$, we get

$$\begin{aligned} H_{n}\in L(Y, {{\mathcal {P}}}_{2n+1})=(Y{\hat{\otimes }} G)^{*}. \end{aligned}$$

Thus, we can look to $H_{n}$ as a linear functional in $(Y{\hat{\otimes }} G)$. But a bounded linear functional on a Banach space, if it attains its norm, it is attains at some extreme point (see, for example Khalil 1990). Consequently:

### **Lemma 5**

$H_{n}$ *attains its norm at some extreme point of* ${\mathbf{R}}^{n+1}\times {\mathbf{R}}^{n+1}.$

### *Proof*

Follows from the fact that $Ext(E{\hat{\otimes }} F)=Ext(E){\otimes }Ext(F).$ Now, if *E* and *F* are Banach spaces, and $W=E \times F$, with the $\infty -$norm. Then $(x,y) \in W$ is extreme if and only if $\Vert x \Vert =\Vert y\Vert =1$, and $x \in Ext(E), y\in Ext(F)$. But extreme points of ${\mathbf{R}}^{n+1}$ are those of the form $(x_{i})=\mp 1$ for all *i*. Hence $\square$

### **Lemma 6**

$\Vert H_{n}\Vert =\sup \Vert \sum _{i=0}^n v_{i}(x)\ell _{i}^2(x) a_{i}+\sum _{i=0}^n (x-x_{i})\ell _{i}^2(x)b_{i}\Vert$ *where the supremum is taken over all* $a_{i}=\mp 1$ *and* $b_{i}=\mp 1$ *, for all i*.

Also, since $v_{i}(x) \ge 0$ and $\ell _{i}^2(x)>0$, we get

### **Theorem 4**

22 $$\begin{aligned} \Vert H_{n}\Vert =\sup \left( \sum _{i=0}^n v_{i}(x)\ell _{i}^2(x)+\sum _{i=0}^n |x-x_{i}|\ell _{i}^2(x)\right) . \end{aligned}$$

Thus, to estimate (or calculate) $\parallel H_{n}\parallel$ we have to work with (22). A simple consequence of equation (22) is:

### **Theorem 5**

$$\Vert H_{n}\Vert \le 5.$$

### *Proof*

Since $\sum _{i=0}^n v_{i}(x) \ell _{i}^2(x)=1$ and $|x-x_{i}|\ell _{i}^2(x) \le 2 \ell _{i}^2(x)$, using the fact that $\sum _{i=0}^n \ell _{i}^2(x) \le 2$, the result follows, which agrees with Pottinger’s estimate (Pottinger 1976). $\square$

## Conclusions

The fundamental goal of this paper is to prove convergence of the interpolation functions in the $C^2$-norm, where the error estimates are given in terms of the modulus of continuity of the second derivative. Some results agrees with Pottinger’s estimate (Pottinger 1976), and some are considered to be an improvement over those obtained in Al-Khaled and Khalil (2000). We also establish a general theorem on extreme points for Hermite operator.
